# Addressing the distressing: Pancreatic enzyme replacement therapy mitigates abdominal symptoms and weight loss during chemotherapy for advanced pancreatic cancer: A prospective study

**DOI:** 10.1002/ncp.70096

**Published:** 2026-02-06

**Authors:** Pamela N. Klassen, Christina A. Kim, Jessica Kasnik, Michael B. Sawyer, Sunita Ghosh, Vickie Baracos, Vera C. Mazurak

**Affiliations:** ^1^ Department of Agricultural, Food and Nutritional Sciences University of Alberta Edmonton Alberta Canada; ^2^ Paul Albrechtsen Research Institute CancerCare Manitoba Winnipeg Manitoba Canada; ^3^ Department of Internal Medicine, Section of Medical Oncology and Hematology Rady Faculty of Health Sciences Winnipeg Manitoba Canada; ^4^ Nutrition Services, Cross Cancer Institute Edmonton Alberta Canada; ^5^ Department of Oncology University of Alberta Edmonton Alberta Canada; ^6^ Department of Public Health Sciences Henry Ford Hospital Detroit Michigan USA

**Keywords:** nutrition, palliative oncology, pancreatic cancer, pancreatic enzyme insufficiency, pancreatic enzyme replacement therapy, symptom management

## Abstract

**Background:**

Pancreatic enzyme insufficiency (PEI) contributes to symptom burden and malnutrition in advanced pancreatic cancer (aPC). We aimed to evaluate the impact of pancreatic enzyme replacement therapy (PERT) on symptom burden and weight during chemotherapy.

**Methods:**

Patients with aPC who were referred to a dietitian for suspected PEI at the Cross Cancer Institute (Edmonton, Canada) were enrolled. Baseline (BL) PEI symptoms were evaluated prior to PERT initiation; dose was optimized by 1 month. PEI symptom severity was assessed with the Pancreatic Enzyme Insufficiency Questionnaire (PEI‐Q) at BL, reassessed after 1 and 3 months, and compared between BL and first reassessment. Mean weight change from BL to 1 month (percentage per 30 days) was compared with change from 1 to 3 months. Continuous and categorical variables were compared using paired samples t tests and McNemar test, respectively.

**Results:**

Of 29 patients enrolled, 23 initiated PERT and completed ≥1 symptom reassessment. Median reported PERT dose at reassessment was 200,000 USP lipase units/day (IQR 97,200, 300,000). Improvements in mean severity scores for stomach pain (*P* = 0.001) bloating (*P* = 0.049) and stomach noises (*P* = 0.032) were reported at reassessment, with a trend toward improved appetite (*P* = 0.053). Prevalence of moderate/severe PEI decreased (11/23 *vs* 4/23, *P* = 0.020). Weight loss slowed after 1 month (−4.3 ± 4.8%/30 days [BL to 1 month] vs −0.2 ± 3.9%/30 days, *P* = 0.033).

**Conclusion:**

Patients receiving dietitian‐directed PERT showed improved abdominal symptoms and attenuated weight loss after dose optimization, addressing a patient priority for those with aPC.

## INTRODUCTION

Pancreatic enzyme insufficiency (PEI) is prevalent and progressive in patients with advanced pancreatic cancer (aPC), affecting an estimated 72% of all patients with higher prevalence among those with pancreatic head tumours.^1^ PEI causes nutrient malabsorption leading to abdominal and bowel symptoms and represents a significant unaddressed patient concern.^2^, ^3^ The malabsorption and food avoidance caused by PEI contributes largely to malnutrition, which is marked by rapid weight loss and skeletal muscle loss.^4^ Malnutrition is distressing for patients, contributing to reduced quality of life, functional decline, poorer tolerance of cancer‐directed treatment, and ultimately shorter survival.^5^, ^6^, ^7^, ^8^

Oral pancreatic enzyme replacement therapy (PERT) is recommended to treat PEI in aPC.^9^, ^10^, ^11^, ^12^, ^13^ This recommendation is based largely on consensus, as only a small number of heterogenous studies in aPC have been undertaken with varied outcomes, including quality of life, symptoms, survival, or weight change.^14^ Only one study has investigated symptom impact as a primary outcome, reporting improvement in pancreatic pain, bloating/gas, and general digestive symptoms after 3 weeks of receiving PERT in the context of best supportive care in patients not receiving chemotherapy.^15^ Applicability of PERT to patients receiving cancer‐directed therapy remains unknown. Weight maintenance is another key outcome of interest, as the primary effect of PERT is to restore nutrient absorption. Weight loss attenuation with PERT in aPC has been reported in only two of four studies.^16^, ^17^, ^18^, ^19^

As recommendations for PERT in aPC are increasingly implemented in cancer centers, randomized controlled trials to evaluate its impact have become more difficult to justify and recruit to.^20^, ^21^, ^22^ In Alberta, Canada, the prevalence of PERT use among patients with aPC is over 75% (unpublished data). PERT is provided without cost via provincial pharmaceutical coverage, and all patients are supported by a specialized dietitian. In this context, we undertook a prospective observational study to capture the impact of PERT optimization on patient‐reported PEI symptoms in those with aPC who were offered chemotherapy treatment. Our secondary objective was to explore the rate of weight change prior to PERT optimization, compared with post‐PERT optimization.

## MATERIAL AND METHODS

### Patient advisors in study design

A patient and family advisory committee was established consisting of four individuals who helped to identify patient priorities, selected patient‐reported outcome assessment tools, and advised on assessment timing and frequency. They ensured the study did not excessively burden participants and would be feasible for recruitment and retention.

### Study design

**Figure 1:**
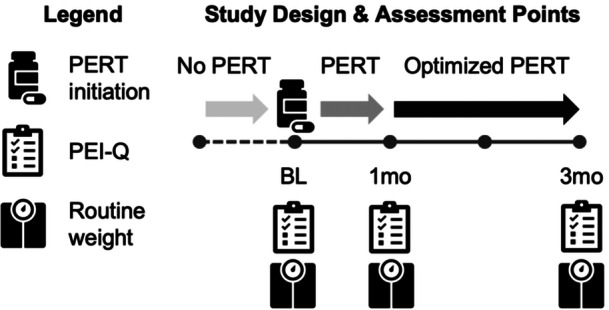
Prospective evaluation of pancreatic enzyme replacement therapy (PERT) impact on patient‐reported symptoms. 1mo, 1 month after PERT initiation; 3 mo, 3 months after PERT initiation; BL, immediately prior to PERT initiation; PEI‐Q, pancreatic enzyme insufficiency questionnaire; PERT, pancreatic enzyme replacement therapy.

All patients with newly diagnosed aPC who were referred to the gastrointestinal oncology dietitian at the Cross Cancer Institute in Edmonton, Alberta, Canada between April 2021 and May 2023 were invited to participate if they met the following inclusion criteria: (1) unresectable (locally advanced or metastatic) pancreatic adenocarcinoma; (2) age ≥18 years; (3) Eastern Cooperative Oncology Group Score of 0–2; (4) life expectancy of ≥2 months in the opinion of the treating medical oncologist; (5) ability to understand and respond to questionnaires in English; (6) suspected by oncologist or dietitian to have PEI. Exclusion criteria included (1) active disease or syndrome causing malabsorption other than pancreatic exocrine insufficiency (ie, short gut, cystic fibrosis, bowel obstruction); (2) uncontrolled hyperglycaemia (random blood glucose of >15.0 mmol/L at screening); (3) inability to swallow capsules; (4) currently using prescription PERT daily of ≥25,000 USP lipase units (USP) per meal or snack; and (5) oral intake <50% of requirements for the past week per dietitian assessment. Exclusion criteria were intended to limit factors other than PERT treatment that could alter malabsorptive symptoms or weight change. All patients were offered standard palliative‐intent chemotherapy at the discretion of their oncologist per usual care. Ethical approval was provided by the Health Research Ethics Board of Alberta – Cancer Committee (HREBA.CC‐20‐0419). Informed consent was obtained from participants after the nature of the study had been explained. As recruitment occurred during a global pandemic, guided remote informed consent was used and questionnaires were sent by email unless the participant requested paper copies by mail. After enrollment, PERT was initiated per standard care at a starting dose of 25,000 USP per meal and snack. The dose was gradually optimized by the dietitian over the first month of therapy to a minimum dose of 40,000 USP per meal and 20,000 USP per snack as per published recommendations by the 1‐month assessment^12^, ^13^, ^23^ (Figure 1). Per standard care for pancreatic cancer, the dietitian also provided nutrition education and counseling to support achievement of protein and energy needs, including recommendations for oral nutritional supplements if appropriate. Regular dietitian follow‐up occurred at least every 2 weeks.

### Study assessments

**Figure 2:**
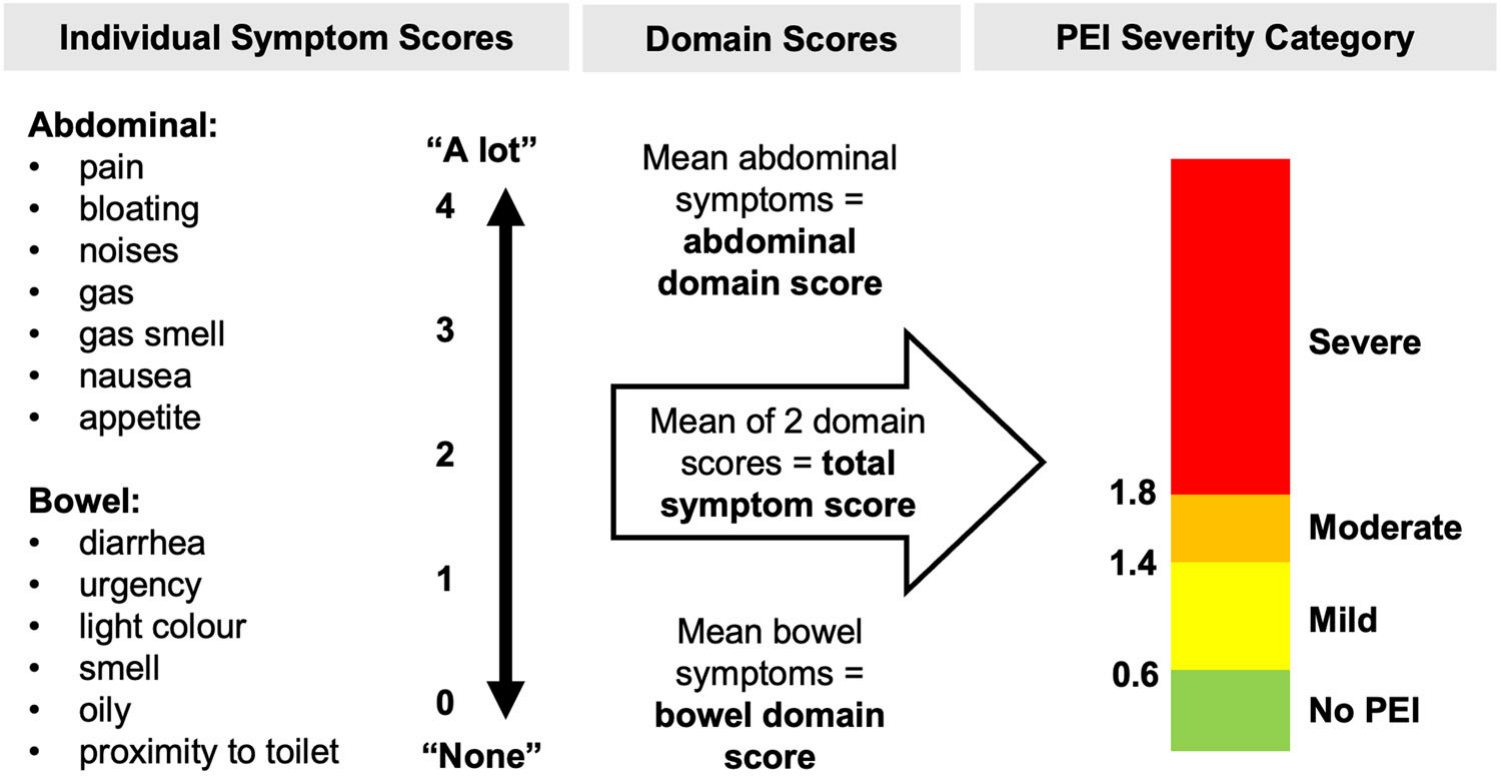
Pancreatic Enzyme Insufficiency Questionnaire (PEI‐Q) scoring.

At baseline (BL), demographic, clinical, pathologic, and treatment characteristics were captured from the clinical record. At the time of each study visit (BL, 1 month, 3 months), patients self‐reported PERT use, including brand, capsule size, and estimated number of capsules taken daily over the past week. Study visits were scheduled to avoid the 7 days after a chemotherapy infusion, reducing confounding by chemotherapy‐induced side effects. The Pancreatic Exocrine Insufficiency Questionnaire (PEI‐Q; Abbot) was used to assess PEI symptoms consisting of 13 questions assessing abdominal and bowel symptoms over the prior 7 days, rated from 0 (none) to 4 (a lot) (Figure 2). Mean abdominal domain score and mean bowel domain score were calculated separately; total symptom score was the mean of the two domain scores. Total symptom scores were categorized into no PEI, mild PEI, moderate PEI, and severe PEI according to the PEI‐Q user manual (Figure 2).^24^

Weight at each study visit was recorded from routine clinic weight measurements if available; missing data were not imputed. Weight change from BL to 1 month and from 1 month to 3 months was calculated as relative change per 30 days (percentage per 30 days) to enable comparison between periods.

### Statistical analysis

The primary outcome was change in symptom scores from BL to first reassessment and included only patients who reported using PERT at their first reassessment. Mean individual symptom score, abdominal domain score, bowel domain score, and total symptom score were compared between BL and the first reassessment using paired *t* tests. Categorical improvement in PEI severity was defined as improvement in PEI‐Q severity by at least one category (eg, severe to moderate, moderate to mild, mild to none). The proportion of patients with no or mild PEI at each time point were compared with exact McNemar test, and the proportion of patients who experienced categorical improvement in PEI severity were compared by BL severity (none or mild *vs* moderate or severe) using chi‐square test. Mean rate of weight change from BL to 1mo and 1 month to 3 months were compared using paired *t* tests.

## RESULTS

### Participants

**Figure 3:**
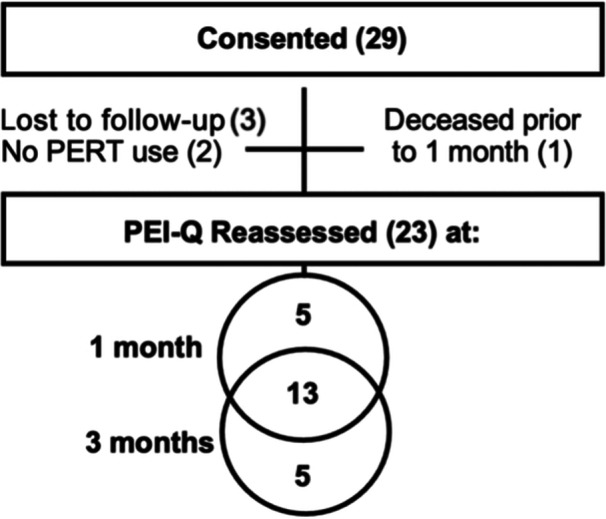
Flow diagram indicating patients consented (*n* = 29) and included (*n* = 23) in primary outcome analysis of symptom change from baseline to first reassessment. First reassessment occurred at 1 month for 18 participants and 3 months for 5 participants. PEI‐Q, pancreatic enzyme insufficiency questionnaire; PERT, pancreatic enzyme replacement therapy.

Twenty‐nine participants consented and completed baseline assessments; 23 completed at least one reassessment while using PERT. Thirteen participants completed reassessments at both 1 month and 3 months (Figure 3). Among those included, 57% had locally advanced disease at BL, and 61% received gemcitabine plus *nab‐*paclitaxel chemotherapy (Table 1). Overweight or obese body mass index was common at BL, despite nearly three‐quarters of respondents reporting >6% body weight loss over the prior 6 months. Although all patients had been referred to the dietitian for suspected PEI and were prescribed PERT per usual local practice based on clinical assessment, three (13%) did not meet the threshold for PEI according to the PEI‐Q total symptom score at BL.

**Table 1:** Baseline characteristics of participants with symptom reassessment.

Characteristic	*N* = 23
Age, years, mean (SD)	68 (12)
Sex, *n* (%)	
Male	13 (56.5)
Female	10 (43.5)
Primary tumour location, *n* (%)	
Head/neck	10 (43.5)
Body/tail	8 (34.8)
Overlapping/unknown	5 (21.7)
Disease stage, *n* (%)	
Locally advanced	13 (56.5)
Metastatic/recurrent	10 (43.5)
Regimen initiated, *n* (%)	
FOLFIRINOX	6 (26.1)
GEM/NAB	14 (60.9)
None (delayed/declined)	3 (13.0)
BMI, kg/m^2^, mean (SD)	26.4 (4.9)
WHO BMI Category, *n* (%)	
<18.5 Underweight	1 (4.3)
18.5–24.9 Normal	8 (34.8)
25.0–29.9 Overweight	9 (39.1)
≥30.0 Obese	5 (21.7)
Reported weight loss from usual, *n* (%)	
<2.5% of usual	3 (13.0)
2.5%–5.9% of usual	3 (13.0)
6.0%–10.9% of usual	5 (21.7)
11.0%–14.9% of usual	4 (17.4)
>15.0% of usual	8 (34.8)
PEI Severity on PEI‐Q, *n* (%)	
No PEI	3 (13.0)
Mild	9 (39.1)
Moderate	7 (30.4)
Severe	4 (17.4)
Proton pump inhibitor use, *n* (%)	
Yes	13 (56.5)
No	9 (39.1)
Unknown	1 (4.3)

Abbreviations: BMI, body mass index; FOLIFIRINOX, multiagent chemotherapy consisting of 5‐fluorouracil, folinic acid, irinotecan, and oxaliplatin; GEM/NAB, doublet chemotherapy consisting of gemcitabine and nab‐Paclitaxel; PEI, pancreatic enzyme insufficiency; PEI‐Q, pancreatic enzyme insufficiency questionnaire.

### PERT use and symptoms

**Figure 4:**
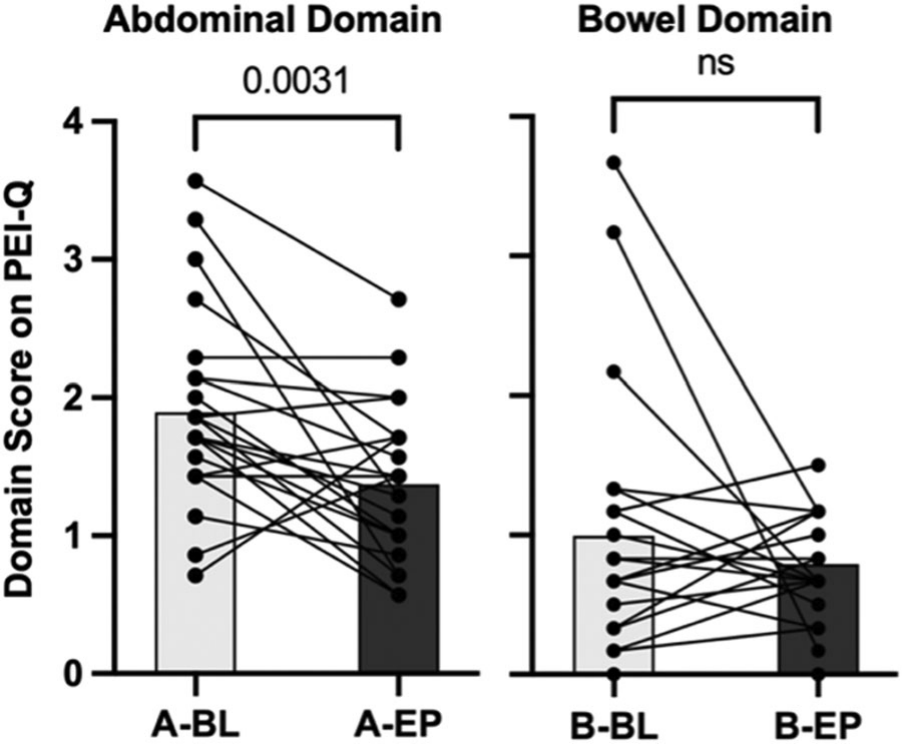
Abdominal (A) and bowel (B) domain scores pre‐initiation and post‐initiation of PERT. Each line represents the change in domain score reported by a single patient between baseline (pre‐PERT) and end point (first reassessment). Bars represent mean domain score at each time point for *n* = 23 participants. Higher scores indicate greater severity. BL, baseline; EP, end point/first reassessment; PERT, pancreatic enzyme replacement therapy.

The primary outcome analysis (change in symptoms from BL to first reassessment) was based on PEI‐Q scores at 1 month for 18 patients and at 3 months for five patients who missed the 1‐month reassessment because of hospitalization or inability to contact. At BL (pre‐PERT), the highest mean symptom scores were in the abdominal domain, including passing gas, stomach noises, stomach pain, and very bad gas smell. Mean bowel domain symptom scores were low at BL. In the week prior to first reassessment, median patient‐reported PERT dose was 200,000 USP/day (IQR: 97,200, 300,000). Overall, abdominal domain score improved significantly from BL to first reassessment (*P* = 0.003), whereas bowel domain score did not change (Figure 4). Individual scores for stomach pain, bloating, and stomach noises improved significantly at first reassessment (all *P* < 0.05) (Table 2). Improvement in appetite approached significance (*P* = 0.053).

**Table 2:** Symptom score change on PEI‐Q from baseline to first reassessment.

Symptom	Baseline *n* = 23	Reassessment *n* = 23	*P* value
Stomach pain	2.1 (1.0)	1.1 (0.8)	**<0.001**
Bloating	1.8 (1.1)	1.3 (0.9)	**0.049**
Stomach noises	2.3 (1.1)	1.6 (1.1)	**0.032**
Passing gas	2.4 (0.9)	2.0 (1.1)	0.272
Very bad gas smell	2.0 (1.2)	1.6 (1.2)	0.224
Nausea	1.2 (1.1)	0.9 (1.0)	0.247
Lack of appetite	1.6 (1.3)	1.12 (1.1)	**0.053**
**Mean abdominal domain**	1.9 (0.7)	1.4 (0.6)	**0.003**
Diarrhea	0.9 (1.2)	1.1 (1.0)	0.478
Bowel urgency	0.9 (0.9)	0.8 (0.8)	0.692
Light/orange stool	1.2 (1.3)	1.0 (1.0)	0.443
Very bad smelling stool	1.6 (1.3)	1.2 (1.0)	0.304
Visible oil in stool	0.7 (1.2)	0.4 (0.6)	0.213
Need proximity to toilet	0.7 (0.9)	0.3 (0.4)	0.083
**Mean bowel domain**	1.0 (0.9)	0.8 (0.4)	0.318

*Note*: Scores are mean (SD).

Abbreviation: PEI‐Q, pancreatic enzyme insufficiency questionnaire.

### Categorical change in PEI severity

**Figure 5:**
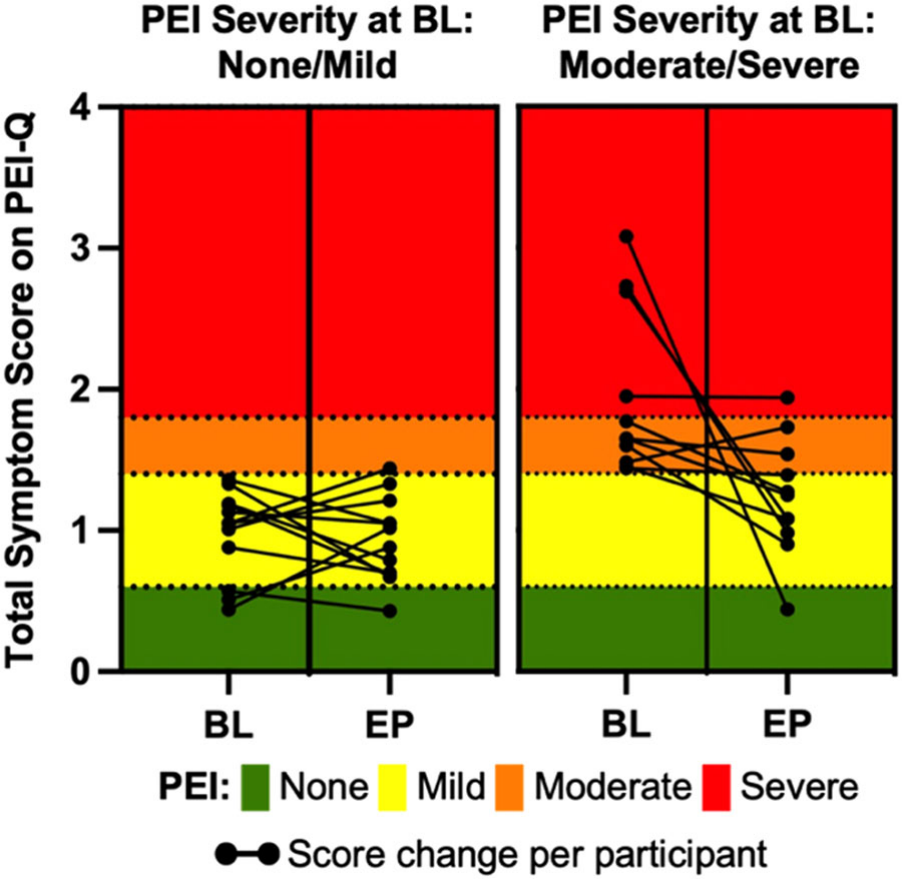
Change in total symptom score and PEI severity category between baseline (pre‐PERT) and end point (first reassessment), by baseline severity. Colors represent severity category. Lines indicate total symptom score change for individual patients 12 participants had none/mild PEI at baseline and 11 participants had moderate/severe PEI at baseline. BL, baseline; EP, end point/first reassessment; PEI, pancreatic enzyme insufficiency; PEI‐Q, pancreatic enzyme insufficiency questionnaire; PERT, pancreatic enzyme replacement therapy.

According to the severity categories of the PEI‐Q, 8 out of 23 patients (35%) demonstrated categorical improvement in overall symptom severity at the first reassessment (Figure 5). There was a significant reduction in the prevalence of moderate/severe PEI between BL and first reassessment (11/23 vs 4/23, *P* = 0.020). Categorical improvement was more prevalent among patients with moderate or severe PEI at BL compared with those with no PEI or mild PEI at BL (8/11 *vs* 0/12, *P* < 0.001).

### Weight change

As a secondary outcome, repeated weight measurements at BL, 1 month, and 3 months were available for as subset of 15 patients. Significantly greater weight loss was observed from BL to 1 month vs 1 month to 3 months (−4.3 ± 4.8%/30 days vs −0.2 ± 3.9%/30 days, *P* = 0.033). Among these participants, 14 out of 15 had stable disease or partial response to therapy at 3 months. Mean abdominal symptom score for those with 3‐month PEI‐Q data in the subset was 2.0 ± 0.6 at BL, which reduced to 1.2 ± 0.4 at 3 months (*P* = 0.005, *n* = 12). Mean bowel score was low at BL and remained unchanged at 3 months in this subset (1.1 ± 0.8 vs 1.1 ± 0.6, *P* = 0.965).

## DISCUSSION

### Impacts of PERT

This study confirms that abdominal symptoms of PEI are a prevalent concern for patients with aPC.^2^ Stomach pain, bloating, stomach noises, passing gas, gas smell, and poor appetite were the top six worst abdominal symptoms in patients presenting with a new diagnosis of aPC with suspected PEI. After initiating PERT, significant improvements in stomach pain, bloating, and stomach noises were observed, as well as a trend toward improved appetite. Overall, abdominal symptoms were substantially reduced with initiation of PERT. These results are consistent with a prior report by Landers et al. in which PERT initiation resulted in reduced pancreatic pain and bloating/gas symptoms after 3 weeks in patients who were not receiving cancer‐directed treatment.^15^ We have herein shown that the benefits with PERT are also seen in those receiving chemotherapy.

In contrast to abdominal symptoms, bowel symptoms were mild at BL for the majority and did not significantly change, either individually or as a domain. It is possible that this lack of improvement is due to low bowel symptoms scores at BL. Notably, for the three patients whose bowel domain scores were ≥2 at BL, bowel domain scores did improve markedly with PERT. Another possibility may be that those with mild bowel symptoms were experiencing another cause, such as bile acid malabsorption, which would not resolve with PERT. Reasons for low bowel symptom scores at BL in this cohort are unknown. Patients with severe bowel symptoms prior to presentation at medical oncology may have been given PERT by their family physician or diagnosing surgeon, and thus they were not eligible for this study. Alternatively, some patients may have been prescribed opioid pain medication prior to BL, which can cause constipation or at least offset steatorrhea/diarrhea. Finally, many patients with undiagnosed PEI report intolerance to high‐fat or “rich” foods, and therefore naturally exclude these foods from their diet to prevent bowel symptoms. We suspect that a combination of these factors resulted in low bowel symptoms at BL in our cohort.

Despite significant improvement in abdominal symptoms, only 8 out of 23 patients (35%) demonstrated categorical improvement in overall PEI severity (as per PEI‐Q categories). Yet, among those with moderate or severe PEI at BL (*n* = 11), 70% experienced categorical improvement. In the validation of the PEI‐Q, Johnson et al. specifically noted that “longitudinal data from an intervention sample will be needed to establish clinically meaningful change thresholds in PEI‐Q scores and generate evidence to support the ability of PEI‐Q scores to detect changes over time.”^25^ It is possible that PEI‐Q categorical severity (combining abdominal and bowel domains) is not sensitive enough to detect clinically meaningful change in cases in which one domain score is low at BL; this requires further investigation. Studies of PEI related to chronic pancreatitis also indicate that complete resolution of maldigestion is difficult to achieve even with PERT, making it unsurprising that patients with mild symptoms at BL may not experience complete resolution (reviewed by de la Iglesia‐Garcia et al.^24^). We consider it a positive outcome that PERT is clearly effective for reducing the most prominent symptoms, and for reducing overall severity among those with moderate or severe PEI at BL.

Beyond PERT's impact on symptoms, weight loss was mitigated once PERT dose was optimized at 1 month, suggesting a delayed but significant impact of PERT on weight change. After PERT initiation, patients were instructed to gradually titrate their dose to recommended levels, resulting in improved symptoms by the 1‐month assessment. Resolution of symptoms suggests improved nutrient absorption.^24^ Furthermore, resolution of symptoms may have allowed patients to increase oral intake—evidenced by the trend toward improved appetite at 1 month—further improving energy balance. Bye et al. and Bruno et al. have previously demonstrated the link between symptoms and energy intake.^16^, ^26^ Although we did not collect oral intake data, these patients were uniformly supported by a dietitian who not only directed PERT but provided nutrition advice and therapy to optimize oral intake. We suggest that a combination of improved absorption and improved intake was synergistic in attenuating weight loss from 1 month to 3 months. The assessment of symptoms alongside oral intake in future studies would be required to confirm this hypothesis. Furthermore, Whitcomb et al. specifically identify muscle mass gain as a measure of successful treatment with PERT.^13^ Weight change is impacted by fluctuations in fluid (such as presence of ascites) and adipose tissue, whereas skeletal muscle loss is a more specific hallmark of malnutrition in cancer. We recently demonstrated in a retrospective population‐based cohort that PERT dose was a key factor associated with maintenance of skeletal muscle among patients with PEI; however, this has not been evaluated prospectively.^4^ To confirm PERT's impact on skeletal muscle specifically, a prospective study would require skeletal muscle measurements that clearly delineate a pre‐PERT period and a PERT‐optimized period for comparison of muscle change.

### Strengths, limitations, and challenges

In this patient‐oriented study, our primary outcome was based on a standardized patient‐reported symptom assessment tool specific to PEI, as opposed to clinician‐reported or generic cancer symptom assessments. Further, the tool evaluated symptom change on a continuous scale rather than dichotomizing symptom presence or absence.^27^ Repeated measures design strengthened internal validity, eliminating the impact of interindividual variability on our outcome as each patient acted as their own control. Limitations stem from the study's single‐arm design, in which the lack of a control group made it difficult to infer causation. Given the lack of clinical equipoise to randomly assign patients with aPC and suspected PEI to receive a PERT placebo, this could not be ethically avoided. Disease response to treatment could also result in improved symptoms and weight stabilization, and thus it is not possible to discern whether the benefits relate to PERT alone. We used the first reassessment time point for primary outcome analysis, which was at 1 month for 18 out of 23 patients, to reduce the time between PERT initiation and reassessment, thereby limiting the opportunity for other factors to affect symptoms. Another limitation is related to potential underrecognition of bile acid malabsorption, which could cause similar symptoms to PEI but would not resolve with PERT. Although biliary obstruction is routinely managed with stenting at our center, assessment and treatment of bile acid malabsorption is not common. Finally, we must consider the possibility that weight stability after 1 month on PERT could have been influenced by fluid shifts such as ascites, which is common in this population.

The study experienced challenges to recruitment and retention, as have been reported by others.^22^ Recruitment was initiated in early 2021 while a global pandemic was impacting in‐person medical care and diagnostic imaging, which may have caused delays in cancer diagnoses and consultations.^28^, ^29^ Nonresponse at one of the two reassessments was often due to patients admitted to hospital or those unable to access the required technology. All considered, attrition of 21% (6 out of 29) was comparable to other studies in aPC.^15^, ^30^

## CONCLUSION

PERT has been increasingly recommended to support nutrition optimization and symptom management in people with aPC, but evidence of benefit during cancer‐directed treatment was previously limited. Our results affirm the importance of PERT, demonstrating clear benefits on patient‐centered outcomes, including reduction in abdominal symptoms and attenuated weight loss. Availability of PERT therapy internationally often depends on the ability of patients to pay or to access private insurance.^31^ Given mounting evidence that PERT impacts outcomes that matter to patients, action and advocacy are required to ensure that this helpful care strategy is available to all who require it.

## AUTHOR CONTRIBUTIONS

Pamela N. Klassen, Christina A. Kim, Jessica Kasnik, Michael B. Sawyer, and Vera C. Mazurak equally contributed to the conception and design of the research. Sunita Ghosh contributed to the design of the research. Pamela N. Klassen, Sunita Ghosh, Vickie Baracos and Jessica Kasnik contributed to the acquisition and analysis of the data. Pamela N. Klassen, Michael B. Sawyer, Vickie Baracos and Vera C. Mazurak contributed to the interpretation of the data. Pamela N. Klassen and Christina A. Kim drafted the manuscript. All authors critically revised the manuscript, agree to be fully accountable for ensuring the integrity and accuracy of the work, and read and approved the final manuscript.

## CONFLICT OF INTERESTS STATEMENT

Vickie Baracos is a consultant for Pfizer and Nestle Health Science. Michael B. Sawyer and Jessica Kasnik have previously been engaged as speakers and advisors for Viatris Canada. Christina A. Kim has received research funding from Celgene Inc. and speaker honoraria from Amgen, both unrelated to this work. These agencies had no role in the funding or design of the study; in the collection, analyses, or interpretation of data; in the writing of the manuscript; or in the decision to publish the results. The remaining authors declare no conflicts of interest.
